# Predictive Value of Proteinuria in Adult Dengue Severity

**DOI:** 10.1371/journal.pntd.0002712

**Published:** 2014-02-20

**Authors:** Farhad F. Vasanwala, Tun-Linn Thein, Yee-Sin Leo, Victor C. Gan, Ying Hao, Linda K. Lee, David C. Lye

**Affiliations:** 1 Department of Family Medicine and Continuing Care, Singapore General Hospital, Singapore; 2 Communicable Disease Center, Tan Tock Seng Hospital, Singapore; 3 Yong Loo Lin School of Medicine, National University of Singapore, Singapore; 4 Saw Swee Hock School of Public Health, National University of Singapore, Singapore; Tropical Medicine Institute Pedro Kourí, Cuba

## Abstract

**Background:**

Dengue is an important viral infection with different presentations. Predicting disease severity is important in triaging patients requiring hospital care. We aim to study the value of proteinuria in predicting the development of dengue hemorrhagic fever (DHF), utility of urine dipstick test as a rapid prognostic tool.

**Methodology and principal findings:**

Adult patients with undifferentiated fever (n = 293) were prospectively enrolled at the Infectious Disease Research Clinic at Tan Tock Seng Hospital, Singapore from January to August 2012. Dengue infection was confirmed in 168 (57%) by dengue RT-PCR or NS1 antigen detection. Dengue cases had median fever duration of 6 days at enrolment. DHF was diagnosed in 34 cases according to the WHO 1997 guideline. Dengue fever (DF) patients were predominantly younger and were mostly seen in the outpatient setting with higher platelet level. Compared to DF, DHF cases had significantly higher peak urine protein creatinine ratio (UPCR) during clinical course (26 vs. 40 mg/mmol; p<0.001). We obtained a UPCR cut-off value of 29 mg/mmol based on maximum AUC in ROC curves of peak UPCR for DF versus DHF, corresponding to 76% sensitivity and 60% specificity. Multivariate analysis with other readily available clinical and laboratory variables increased the AUC to 0.91 with 92% sensitivity and 80% specificity. Neither urine dipstick at initial presentation nor peak urine dipstick value during the entire illness was able to discriminate between DF and DHF.

**Conclusions:**

Proteinuria measured by a laboratory-based UPCR test may be sensitive and specific in prognosticating adult dengue patients.

## Author Summary

Dengue infection is getting more common in recent years, affecting all age groups. Currently, there is no specific treatment for dengue. Close clinical monitoring and careful fluid therapy is the only way of management for those with severe dengue disease, i.e., dengue hemorrhagic fever (DHF) or dengue shock syndrome (DSS). It is crucial to know among patients with dengue, who will potentially progress to DHF/DSS in the most reliable, economical and fastest way, so as to prioritise limited resources. We investigated the presence of protein in urine as an indicator of progression to DHF/DSS. Adult patients with dengue were enrolled to our clinic. Clinical data, blood and urine were collected. We found that patients who were going to develop DHF/DSS had increased protein one day prior to developing to DHF/DSS. DHF/DSS cases had higher protein levels in urine compared to patients with just dengue fever. Laboratory-based urine protein data, when used together with other readily available blood tests, helped to detect 92% of DHF cases correctly. Currently available clinic-based urine protein test strip was not useful in predicting severe disease. Future studies may improve the ability of the clinic-based tests, thus reducing the reliance on laboratory testing.

## Introduction

Dengue is an important arthropod-borne disease affecting millions of people in tropical and subtropical regions and is the most prevalent mosquito-borne viral disease in South East Asia with significant morbidity and mortality [1]–[3]. It is caused by the four dengue virus strains transmitted by the *Aedes* mosquito. Risk of severe disease and death especially in children underscores the importance of early detection of dengue fever (DF) and monitoring for signs of progression to severe disease namely dengue hemorrhagic fever (DHF) or dengue shock syndrome (DSS) [4].

Several studies have attempted to combine clinical and simple laboratory tests to predict DHF/DSS. A probability equation and decision tree incorporating clinical bleeding, hypoproteinemia, lymphopenia and elevated serum urea were derived and validated in adult DHF in Singapore [5]–[7]. While these are promising, the need for serum protein and urea reduces its utility in resource-limited settings. An algorithm incorporating leukocyte, monocyte and platelet counts with serum hematocrit predicted pediatric DSS in Thailand [8].

Our previous series of hospitalized patients with dengue showed that the onset and peak of proteinuria using the urine protein creatinine ratio (UPCR) was associated significantly with the development of DHF. The small hospitalized cohort comprised mostly DHF patients, as patients with DF are mostly treated in the community. We postulated that the degree of proteinuria may indicate the severity of dengue infection. The significant peak proteinuria could be a manifestation of a pathogenic mechanism that the virus triggers on the lymphoreticular system, resulting in glomerular leakage of protein associated with DHF [9], [10].

A recent Vietnamese study on febrile children showed that the urine albumin creatinine ratio (UACR) was higher in dengue patients compared to patients with other febrile illnesses, but the discrimination between the two diagnostic groups in the early febrile phase was poor. Secondly, UPCR did not prove useful in predicting either development of warning signs for severe dengue or need for hospitalization [11].Transient proteinuria takes place in patients with febrile illness. However, in the context of a patient diagnosed with dengue fever, we aim to determine in this adult prospective dengue study (1) if the rise in proteinuria in a population group epidemiologically suspected of having dengue can predict the subsequent development of adult DHF or DSS. (2) compare the laboratory measurement of urine protein creatinine ratio with a urine dipstick to explore the latter as a rapid prognostic test, and (3) improve the prognostic accuracy of proteinuria alone in combination with simple and readily available clinical and laboratory data.

## Methods

### Ethics statement

This study was approved by the National Healthcare Group Domain Specific Review Board (DSRB/E/09/00432). Written informed consent was taken from every subject. All data were anonymised.

### Participants

Adult patients (≥18 years) with acute undifferentiated fever were enrolled at the Infectious Disease Research Clinic, Communicable Disease Center (CDC), Tan Tock Seng Hospital (TTSH), Singapore between January to August 2012. Patients were referred from emergency departments or primary care clinics in Singapore. Subjects with pre-existing renal disease were excluded from the analysis. Real-time polymerase chain reaction or non-structural protein 1 antigen tests were performed on patients' sera at Environmental Health Institute (EHI) to confirm dengue infection [12], [13]. Dengue positive patients were followed up daily during the acute period and subsequently 21–30 days from enrolment. Demographic and epidemiological data were collected at enrolment and symptoms and signs were recorded at each visit. Hematology and biochemistry tests were performed daily at the Department of Laboratory Medicine (DLM), TTSH.

### Urine sample collection and testing

Spot urine collection was done by the mid-stream clean-catch method on each study day. Presence of protein in the urine was checked immediately in the clinic using Micral-Test^R^. Urine protein creatinine ratio (UPCR) was performed by DLM to confirm the amount of proteinuria (M-TP and CREm of Beckman Coulter, Inc). All tests were performed according to the manufacturer's instructions. Clinically significant proteinuria was defined as UPCR≥20 mg/mmol in line with criteria from the US National Kidney Foundation [14].

### Clinical outcome

Dengue hemorrhagic fever (DHF) defined according the World Health Organization (WHO) 1997 dengue guideline was the primary outcome of the study. Subjects must have fever or history of fever, hemorrhagic manifestations, thrombocytopenia (platelet ≤100×10∧9/L), and plasma leakage to fulfil DHF. We noted the day that the subjects fulfilled DHF definition. For DSS, DHF cases required either (i) tachycardia with narrow pulse pressure or (ii) systolic blood pressure (SBP) <90 mmHg [4], [15].

### Statistical analysis

For descriptive analyses, median, 5^th^ and 95^th^ percentiles (pctl) were used for continuous variables. Number and percentage were used for categorical variables. Wilcoxon rank sum test with continuity correction was used to compare continuous variables between DF and DHF groups, as well as patients who remained DF throughout versus those without DHF at initial presentation but subsequently developed DHF. Chi-square and Fisher's exact tests were used to compare categorical variables between the same groups. A p-value of less than 0.05 was considered significant.

We studied the cut-off point of UPCR peak value using receiver operating curve (ROC) analysis to obtain the best sensitivity and specificity in differentiating between DF and DHF. Multivariate logistic models were applied to determine whether UPCR in the model can be improved in terms of area under the curve (AUC) by incorporating several readily available clinical and laboratory variables including any bleeding manifestation, white blood cell count, serum hematocrit, platelet count and serum protein. To study the role of UPCR in discrimination of DHF, we used the data available at the time of initial presentation as well as throughout the entire illness.

We assessed trends of UPCR by defervescence day for DF and DHF and trend of UPCR by DHF onset day for the subgroup without DHF at initial presentation but subsequently developed DHF by box plots. The day of defervescence was taken as day 0, and one day before as day −1 and one day after as day +1. In a separate analysis by DHF onset day, the day of DHF was taken as day 0.

In addition, we applied Bayesian modeling with the Markov chain Monte Carlo method to compare the daily mean changes of UPCR between DF and DHF [16]. All statistical analyses were performed in statistical software R or openBUGS [17].

## Results

### Participants

There were 293 subjects recruited from January to August 2012. Dengue PCR or NS1 was detected in 168 (57%). Median age was 34 years age (5^th^–95^th^ pctl 22–55) and 79% were males. Six subjects had co-morbid conditions, namely 1 liver disease, 3 diabetes mellitus and 2 cancers. Twenty nine percent of subjects were treated as inpatient in the entire course of the illness. Among the dengue positive cases, median duration of fever onset until study enrolment was 6 days (5^th^–95^th^ pctl 3–9). Median number of follow up visits during acute illness was 3 days (5^th^–95^th^ pctl 2–6).

### Clinical outcomes

Among the 168 patients, 34 (20%) had DHF, 8 of which were diagnosed with DHF at enrolment visit. The rest of the DHF patients came from the initial cohort of DF patients that progressed to DHF during the course of their illness. Two out of 34 DHF cases had DSS in the whole cohort. There were no fatalities.

### Proteinuria between DF and DHF patients

Eleven subjects (10 DF and 1 DHF) were excluded from further analyses because their very low urine protein levels (<0.1 g/L) rendered their UPCR results invalid. Table 1 showed the detailed differences in UPCR values among DF and DHF patients. DF patients were predominantly younger and were mostly seen in the outpatient setting with higher platelet levels than DHF patients. DHF patients had significantly higher peak levels of proteinuria than DF patients; 85% of DHF cases versus 61% of DF cases had significant UPCR≥20 mg/mmol. Additionally the median UPCR value on the day of initial presentation was significantly higher in patients without DHF initially but subsequently developed DHF versus DF patients. Both groups of patients developed maximum proteinuria at median of 7^th^ day of illness. In Table 2 neither urine dipstick at initial presentation nor peak urine dipstick value during the entire illness was able to discriminate between DF and DHF. Semi-quantitative dipstick test results were shown in supplementary table S1.

**Table 1:** Patient characteristics and laboratory parameters.

Dengue positive sub-group	DF (n = 124)	DHF (n = 33)	p value
Age, years	34(22–44)	43(22–60)	**<0.001**
Male, n (%)	104(84)	25(76)	0.41
Inpatients, n (%)	26(21)	23(70)	**<0.001**
**UPCR values**			
UPCR peak value	25.5(10–87)	40(15–251)	<**0.001**
UPCR peak illness day	7(4–10)	7(5–9)	**0.039**
Significant UPCR (≥20 mg/mmol)	76(61)	28(85)	**0.02**
Significant UPCR onset as illness day	7(4–9)	7(5–9)	0.14
UPCR peak value before DHF	-	36(12–250)	**-**
**Corresponding laboratory values at peak UPCR**			
Hematocrit %	44(37–49)	44(37–49)	0.36
Platelet, ×10∧9/Liter	91(43–207)	54(17–123)	**<0.001**

Dengue positive subgroups, excluding DHF at initial presentation	DF (n = 124)	DHF (n = 26)	p value
UPCR peak value	25.5(10–81)	39(16–250)	**0.001**
Initial UPCR value	23(11–81)	33(11–247)	**0.03**
Initial hematocrit %	44(37–49)	43(37–49)	0.51
Initial platelet, ×10∧9/Liter	101(50–191)	81(23–141)	**0.01**

UPCR = urine protein creatinine ratio, DF = dengue fever, DHF = dengue hemorrhagic fever.

For dichotomous variables, numbers of cases are shown with percentages in parenthesis; for continuous variables, median values are shown with 5^th^–95^th^ percentiles in parentheses.

**Table 2:** Urine dipstick test results.

Dengue positive sub-group	DF (n = 134)	DHF (n = 34)	p value
Dipstick test positive at first visit*	68(54)	21(66)	0.35
Dipstick test positive during entire illness	98(73)	29(85)	0.21

DF = dengue fever, DHF = dengue hemorrhagic fever.

Numbers of cases are shown with percentages in parentheses.

P values were calculated by Fisher's exact test.

* Values based on 125 DF and 32 DHF.

**Figure 1:**
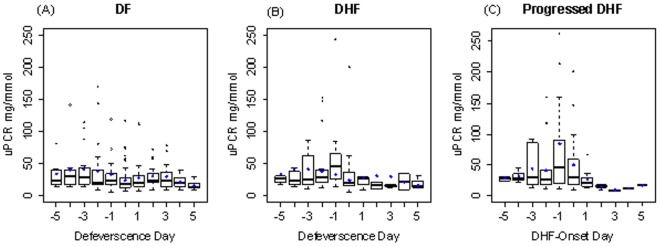
Box plots of daily UPCR for dengue fever (DF) (A), dengue hemorrhagic fever (DHF) before DHF onset by defervescence day (B) and by DHF onset day (C). Black bars in the boxes indicate daily medians whereas blue diamonds indicate daily means of UPCR. The length of whiskers are 1.5 times the interquartile range from the box, the dots which are out of the whiskers are the outliers.

Fig. 1A and 1B show trends of UPCR in DF and DHF patients in relation to day of defervescence (Figure 1A and 1B). Patients in the DHF group showed significantly increased proteinuria one day before the onset of DHF (Figure 1C).

**Figure 2:**
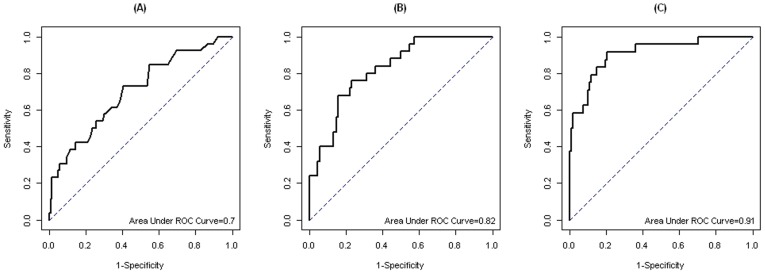
Receiver-operating characteristic curves of prediction of DHF by peak UPCR only (A), the logistic regression model using UPCR at initial presentation adjusted by age and illness day (B) and the logistic regression model using white blood cell count, serum hematocrit, platelet count, serum protein, bleeding and UPCR at initial presentation (C). By maximizing sensitivity and specificity, the peak UPCR cut-off of 29 mg/mmol yielded sensitivity 76% and specificity 60%. From logistic regression model using UPCR at initial presentation adjusted by age and illness day, the maximum sensitivity and specificity were 76% and 76.9%. The maximized sensitivity and specificity of logistic regression model using white blood cell count, serum hematocrit, platelet count, serum protein, bleeding and UPCR at initial presentation were 91.7% and 79.6%.

We obtained a UPCR cut-off value of 29 mg/mmol based on maximum AUC in ROC curves of peak UPCR for DF versus DHF, corresponding to 76% sensitivity and 60% specificity (Figure 2A). The ROC curve logistic regression model using UPCR only (adjusted for age and illness day) indicated good discrimination between DF and DHF as the AUC was 0.82 (Figure 2B). Multivariate analysis with other readily available clinical and laboratory variables (bleeding manifestations, white blood cell count, serum hematocrit, platelet count, serum protein) increased the AUC to 0.91 (Figure 2C).

**Figure 3:**
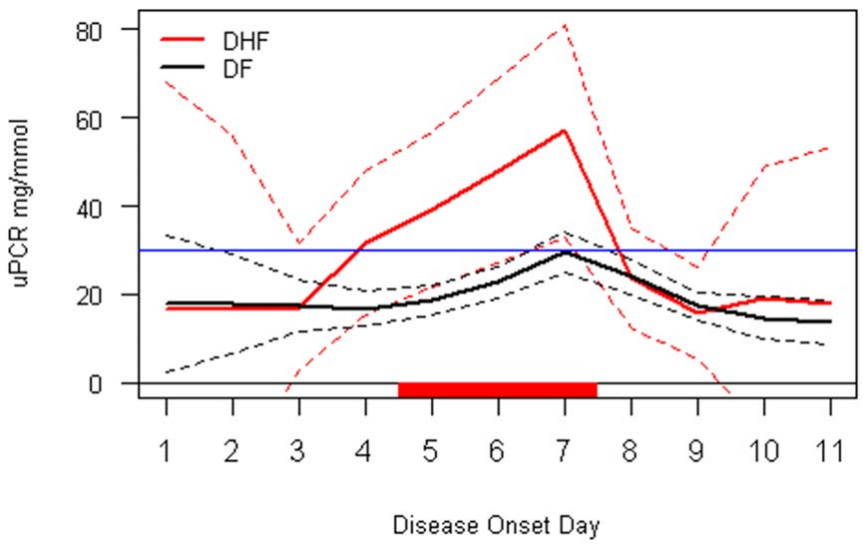
Time course analysis of proteinuria for DF and DHF. Overall means are indicated as solid lines with 95% credible intervals as dashed lines. The red bar on X-axis indicated days with a “significant” difference between DF and DHF. The blue line indicated UPCR level of 29 mg/mmol.

A time course analysis of daily UPCR was presented in Figure 3. Before the third day of illness, the UPCR level in DF and DHF patients were similar. Between days 4 and day 7, the two groups showed significantly different trends of proteinuria. The DHF group showed rapidly increased proteinuria versus DF group, whose proteinuria rapidly decline by day 7.

## Discussion

Dengue hemorrhagic fever and dengue shock syndrome may result in higher morbidity in adult patients. Early prediction of dengue complications may enable close monitoring and improved case management to optimize clinical outcome [18].

Hypoalbuminemia and proteinuria are well recognized in dengue infection [2]. This is postulated to be due to altered filtration of the glycocalyx [19], as dengue virus [20] and NS1 [21] are known to attach to heparan sulphate, which is part of the glycocalyx. Our previous pilot study highlighted difference in proteinuria in hospitalized adult DHF versus non-DHF [10]. Our current prospective study confirmed these findings and further characterized the natural history and utility of proteinuria in discriminating between adult patients with and without DHF. Important differences between our study cohort and that in Hanh Tien's [11] are that the latter studied children in first 3 days of illness, measured microalbuminuria rather than proteinuria, and compared against different outcomes of other febrile illnesses, platelet nadir, hemoconcentration, warning signs and hospitalization.

Notably, using UPCR adjusted for age and illness day had a sensitivity of 76% and specificity of 77% for predicting DHF, while combination it with white blood cell count, serum hematocrit, platelet count, serum protein and bleeding had a sensitivity of 92% and specificity of 80%. This compared favorably with other investigational biomarkers as well as predictive algorithms (Table 3). Commercially available urine dipsticks were not useful as a prognostication tool in our study. Nonetheless, perhaps test strips could be developed to differentiate UPCR values based on our discriminatory cut-off value of 29 mg/mmol.

**Table 3:** Comparison of performance of various reported biomarkers and predictive algorithms for dengue hemorrhagic fever.

Study	Sensitivity	Specificity	Positive predictive value	Negative predictive value
Soluble tumour necrosis factor receptor 80 [23]	67	80	66	69
Soluble tumour necrosis factor receptor 75 [24]	93	34	27	95
Dengue viral load [25]	-	-	88	95
Free secreted non-structural protein 1 [26]	72	79	81	69
Platelet IgM [27]	49	92	-	-
Predictive equation (bleeding, lymphopenia, serum protein and urea) [5]	98	60	10	99
Decision tree (bleeding, serum protein and urea) [6]	100	46	8	100
Cytokines, protein adducts, serum proteins, clinical features [28]	83	75	-	-
Urine protein creatinine ratio, white cell and platelet count, serum hematocrit and protein, bleeding (present study)	92	80	-	-

In our current study involving adult dengue patients, we observed that the peak UPCR could distinguish patients likely to develop DHF from those who did not and that peak UPCR occurred at day 7 of the illness. A significant increase in proteinuria was seen one day before defervescence which corresponded to one day before the development of DHF. Patients with uncomplicated DF had significantly lower proteinuria than patients with impending DHF and DSS. Daily follow-up in this prospective study enabled a time course analysis showing that the discriminatory value of proteinuria was not evident in the early febrile period but it is discriminatory between days 4 and 7, just before defervescence when maximal plasma leakage classically occurs.

One limitation of this study was the small number of DHF patients. Moreover, our study excluded patients aged less than 18 years. Hanh Tien et al showed that a point-of-care test using UPCR would not be sufficiently robust to be useful for early diagnosis and risk prediction in dengue endemic areas [11]. This is in contrast with our study, which uses a hospital-based UPCR which may have better sensitivity and specificity compared with point-of-care UPCR kits. Another limitation is that no single diagnostic assay in isolation is adequately sensitive and specific enough to diagnose all acute cases of dengue. We used either RT-PCR or NS1 structural protein to diagnose patients with dengue. RT-PCR is a robust test during the viraemic febrile phase, but is less sensitive during the time of the defervescence. NS1 rapid diagnostic tests have 49.4–98.9% sensitivity and 90.6–100% specificity in the detection of dengue ranging from 1–15 days of illness in the recent review [22].

### Conclusion

We conclude that the onset and peak proteinuria using a laboratory-based UPCR test was significantly associated with subsequent development of DHF in both ambulatory and hospitalized adult dengue patients. Daily UPCR may be a useful, sensitive and specific prognostic tool in conjunction with clinical parameters to help triage patients requiring hospital care.

## Supporting Information

Checklist S1 STROBE checklist for the reporting of observational studies.
(DOC)

Table S1 Semi-quantitative urine dipstick test results for dengue positive subgroup.
(DOC)
