# Supplementary material for: Predictive Value of Proteinuria in Adult Dengue Severity
Source: PLoS Negl Trop Dis. 2014 Feb 20;8(2):e2712. doi: 10.1371/journal.pntd.0002712 (PMC3930505; doi:10.1371/journal.pntd.0002712)
Supplement: Table S1 — Semi-quantitative urine dipstick test results for dengue positive subgroup. (DOC) [file pntd.0002712.s002.doc]

Table S1. Semi-quantitative urine dipstick test results for dengue positive subgroup

|  | **DF (n=125)** | **DHF (n=32)** | **p value** |
| --- | --- | --- | --- |
| **At first visit** |  |  | 0.32 |
| Negative | 57 (45.6%) | 11 (34.4%) |  |
| Ca.20 | 35 (28.0%) | 9 (28.1%) |  |
| Ca.50 | 23 (18.4%) | 6 (18.8%) |  |
| Ca.100 | 10 (8.0%) | 6 (18.8%) |  |
|  | **DF (n=134)** | **DHF (n=34)** | **p value** |
| **Peak value during illness** |  |  | 0.24 |
| Negative | 36 (26.9%) | 5 (14.7%) |  |
| Ca.20 | 44 (32.8%) | 12 (35.3%) |  |
| Ca.50 | 35 (26.1%) | 8 (23.5%) |  |
| Ca.100 | 19 (14.2%) | 9 (26.5%) |  |

DF=dengue fever, DHF=dengue hemorrhagic fever

Numbers of cases are shown with percentages in parentheses

P values were calculated by chi-square test

Ca.20, Ca.50, Ca.100 represent detecting urinary albumin at 20, 50, 100 mg/L respectively using Micral-Test^R^
